# Dr. Vaughan, on the Danger of Introducing Unknown Poisons

**Published:** 1799-07

**Authors:** Walter Vaughan

**Affiliations:** Rochester


### *To the Editors of the Medical and Phyfical Journal.*

GENTLEMEN,

HAVING perufed, with great fatisfaction, the three firft numbers of your Journal, my inclination leads me to fend you a cafe which fell under my obfervation, in April laft: this cafe, as demonftrative perhaps of the danger of of introducing unknown poiſons into the human ſyſtem, may not be unacceptable to ſome of your readers.

I am,

Gentlemen,

ROCHESTER. Your obedient Servant,

*May* 21, 1799. WALTER VAUGHAN.

Mr. CHARLES GREEN, ſadler at Northfleet, a man about thirty-five years old, athletic, and convivial, being deſirous of loſing ſome blood, conſulted his ſurgeon, and was aſſured by him, that it was improper,—if for no other reaſon,—becauſe unneceſſary. This honeſt aſſurance he unluckily did not duly regard: for he applied on the enſuing Sunday to a barber, who, as requeſted to do, opened a vein *(Vena mediana)*. The wound of the vein was very large; and the diſcharge of blood from it was profuſe, quick, and difficult to be ſtopped: when ſtopped, however, the arm was kept quiet, and no pain was felt in it all the next day, nor, indeed, till Tueſday evening. At this time, a pain was felt at the wound particularly, but extending from it as high as the middle of the arm. The pain encreaſing, Mr. Green ſoon began to experience ſome pain of the head, and ſome confuſion of thought, which, together with extreme anxiety, reſtleſſneſs, ſhortneſs of breath, and frequent rigors, made him declare to his wife, his apprehenſion that his having been bled would preſently coſt him his life.

His ſurgeon was ſent for, the firſt time, on Wedneſday morning, to whom the ſides of the vein did not appear to be united; indeed, they appeared as if they had inflamed and ſuppurated; and, about the middle of the arm, there was a tumor almoſt as large as a hen's egg, and very tender to the touch. Mr. Green's pulſe was found ſmall, quick, and irregular; his ſkin was very hot; and there was a ſlight delirium, with ſlight *ſubſultus tendinum*. The tongue was dry, and a little diſcoloured. A cataplaſm of bread and milk was accordingly applied to the wound, and to the tumor; a bliſter was fixed between the ſhoulders, and a gentle purgative medicine was adminiſtered; alſo miſtura camphorata, with æther vitriolicus, was given occaſionally. But, notwithſtanding the bliſter excited a very conſiderable diſcharge, the bowels were ſoluble, and the perſpiration free and general; the ſymptoms (delirium, and ſubſultus tendinum eſpecially) became worſe every hour.

I was therefore requeſted, on Thurſday morning, to conſult with his ſurgeon, and to determine whether any thing further could be done. I then gave it as my opinion, that Mr. Green's ſymptoms proceeded from ſome morbid poiſon, I knew not what, unfortunately introduced by the barber's lancet lancet; but of this no direct evidence could be obtained:—the barber, doubtlefs, fuppofed his lancet to be clean; and he might, perhaps, have forgotten the immediately preceding time and occafion of ufing it.

In the firft place, the barber might have ufed the lancet fome days, or fome weeks before, and in a cafe where no morbid action was going on; for it is a fact incontrovertible, that the healthy fecretions of one man may, under certain circumftances, become poifonous to another; and it is almoft unneceffary to add, that a very fmall quantity of moft morbid poifons, although ever fo much diluted by the fluids of the human body, or by fimple water, is as capable of exciting a difeafe, as the matter containing the poifon is, in its pureft and moft concentrated ftate.

In the fecond place, an abforption had, I thought, taken place, from the inflammation of the abforbents being entirely above the puncture of the vein; the inflamed abforbents were eafily difcovered between the puncture and the tumor, and even for fome way higher than the tumor. Why the poifon caufed a tumor, before it arrived at the axilla, I know not; there are, however, poifons fo acrid as to inflame veffels, while paffing through them, and this was, perhaps, one of them; and may not fome cafual, but unobferved preffure, have ftopped its progrefs to the axilla; as preffure is known to ftop the progrefs of inflammation along the veins?

In the third place, I thought it clear, that fome matter had been abforbed, from the general hiftory of the difeafe, compared with that of the fmall-pox, and of every difeafe produced by the introduction of a morbid poifon through a wound; the wound of the vein underwent inflammation and fuppuration, and then the difeafe appeared. Its appearance fo foon after the application of the poifon, was probably owing to Mr. Green being naturally a healthy man, and to his labouring under no other difeafe at the fame time. How far the weaknefs, induced by the great lofs of blood, may have contributed to the quick apppearance of the difeafe, I leave for others to determine; but that the weaknefs (the diminution of power), bore no proportion to the increafed action, was evinced by the pulfe, the refpiration, the fweat, and every fymptom. Mr. Jones, with his ufual fagacity, faw this at his firft vifit, on Wednefday morning; and his remedies were, in my humble opinion, judicioufly chofen; but he entertained, I believe, no hope of Mr. Green's recovery, from the moment he firft faw him.

The refult of our conference was, that we fhould diminifh the action, or increafe the power of the fyftem. We agreed to repeat the cataplafm, but with the addition of fome unguentum hydrargyri fortius: we agreed alfo to give freely cinchona, opium, and occafionally wine; another blifter was laid laid upon the breast. This was, as already said, Thursday; and on Friday by twelve o'clock at noon, when I saw Mr. Green again, the tumor of his arm had totally subsided; and there were evident marks of inflammation from the bend of the arm to the axilla. But, alas! though Mr. Jones had, in my absence, applied sinapisms to the feet, with a view to relieve the head, yet the disease, which had undoubtedly a regular time of appearing and of ending, went on with such celerity and increase, that Mr. Green died this very day, Friday, in less than three hours after we had left him.

